# Cholera at New Orleans

**Published:** 1849-02

**Authors:** 


### EDITORIAL DEPARTMENT.

*Cholera at New Orleans.*—From a letter by Dr. Wederstrandt, one of the physicians of the charity hospital at New Orleans, addressed to Dr. A. Brigham, of Utica, N. Y., and published in the Boston Med. and Surg. Jour., Jan. 11, 1849, we derive the following items concerning the late visitation of cholera at New Orleans:—The first cases occured on the 12th of Dec. The two persons first attacked were brought from the ship Swanton, just from Havre. The disease broke out on board this vessel at sea, two weeks after its departure, and seventeen persons died before the vessel reached New Orleans. The two cases in progress at its arrival, were brought to the Charity hospital. The next day, it is stated, "numerous cases appeared all over the city, but principally in the houses nearest to the shipping, or among persons employed on the wharves." Since the middle of December, 40 and 50 persons had been admitted daily, up to the date of the letter, Dec. 25th. "Upward of 50 cases have originated in the house among the convalescents from other diseases, and the attendants; three of the washerwomen have taken the disease, and two died."

The duration of the disease has been from three to twelve hours. We quote verbatim the concluding portion of the letter.

"During the first days of the epidemic, nearly all the cases proved fatal; but within the last few days it seems to be rather on the decline, as our admissions and deaths have decreased, and we begin to number many cures, or rather recoveries. We treat the disease on general principles, and according to the indications of each individual case. In the early stage, we have had reason to be satisfied with the preparations of opium and counter-irritants. Some physicians use a large dose of opium and quinine in the beginning, when they get their cases early; they give from thirty to forty drops, and a drachm of tincture of opium, with half a drachm of quinine, for a single dose, and speak highly of their success. In a few cases I have thought that the practice did good, but I have not used it to any great extent. When brought to us, which they generally are, in the cold stage, we use stimulants externally and internally, with nourishing broths, and several have reacted under this treatment, and finally recovered. Males and females, young and old, are alike subjects for this disease; but far more men than women are attacked. We have seen many children die of it, some under five years, and a few old people at a very advanced age. Dr. Watson, in his very interesting and valuable Lectures on the Practice of Medicine, has given a most correct description of the disease as it now prevails among us, and I believe it to be identical with the Asiatic cholera, which he so ably describes.

I remain, very respectfully yours, JOHN C. P. WEDERSTRANDT.

*New Orleans, Dec. 25th,* 1848."

It is stated in a portion of the letter preceding the above quotation, that the ship Swanton left Havre with all the passengers and crew in good health, and that the cholera did not exist at Havre, but some of the passengers were from a part of Germany where cholera was raging.

